# Genetic and Genomic Research on Sweet Potato for Sustainable Food and Nutritional Security

**DOI:** 10.3390/genes13101833

**Published:** 2022-10-11

**Authors:** Yao Xiao, Mingku Zhu, Shaopei Gao

**Affiliations:** 1 Key Laboratory of Sweet Potato Biology and Biotechnology, Ministry of Agriculture and Rural Affairs/Beijing Key Laboratory of Crop Genetic Improvement/Laboratory of Crop Heterosis and Utilization and Joint Laboratory for International Cooperation in Crop Molecular Breeding, Ministry of Education, College of Agronomy & Biotechnology, China Agricultural University, Beijing 100193, China; 2 Institute of Integrative Plant Biology, School of Life Sciences, Jiangsu Normal University, Xuzhou 221116, China

Food security is the main challenge to the developing world, especially in the least developed countries. Among the 7.8 billion global population, about 820 million people experience chronic hunger (www.un.org/, accessed on 9 Jun 2020). According to the 2022 Global Report on Food Crises (GRFC 2022), 193 million people are surviving in acute food insecurity zones across 53 countries/territories. Although in some developed countries, overnutrition rather than undernutrition has been the major public health concern. Nevertheless, from a global perspective, food insecurity and undernutrition are major problems. Furthermore, the ongoing coronavirus disease 2019 (COVID-19) pandemic and Russia-Ukraine conflict will increase this number as developing countries are multiple-hit by disease, hunger, supply chains, economic consequences, and the ban on the export of agricultural commodities.

Over the past several decades, three major cereals, namely rice, wheat, and maize, make up 60% of all staple crops. However, over-reliance on a small number of crop species impacts on the food and nutritional security of the global population, and diversification of crop production is critical for sustainable food systems. One potential solution to these challenges is the utilization of orphan crops, which can diversify crop production, provide more food sources, and contribute to genetic variation. Orphan crops are also known as underutilized, lost, minor, or neglected crops and as crops for the future. Some orphan crops have the advantage of being well adapted to local and regional conditions, even in marginal areas and under adverse environments, require less agricultural inputs, and might be less affected by climate change. Consequently, orphan crops are important for the conservation of agricultural biodiversity and agro-ecosystems which are critical for the long-term sustainability of food and agricultural production.

Sweet potato [*Ipomoea batatas* (L.) Lam] is a dicotyledonous plant that belongs to the morning glory family, Convolvulaceae. After the potato (*Solanum tuberosum* L.) and cassava (*Manihot esculenta* Crantz), sweet potato is the third most important major root and tuber crop in the world. Sweet potato has traditionally been viewed as a “poor man’s crop”, something to be eaten only in times of urgent need such as famine or war, or an “orphan crop”, which has attracted limited attention compared to other staple crops. However, during the last decade, this perception has changed, and it is widely acknowledged that sweet potato has great potential to contribute to the alleviation of malnutrition and hunger in the developing world. It is now generally accepted that sweet potato is an important species for nutrition and food security and as a raw material for the processing of starches, bioethanol, and feeds in various fields. Importantly, sweet potato has an inherent ability to produce more edible energy than most major food crops. Nowadays, sweet potato is considered as a food with high nutritional value, due to exceeding most other staple foods in vitamins A and C, β-carotene, anthocyanins, calcium and dietary fiber. The importance of orange-fleshed sweet potato varieties in supplementing vitamin A deficiencies cannot be overemphasized. Furthermore, sweet potato leaf is also an extremely versatile and delicious vegetable that possesses high nutritional value. Consequently, sweet potato could be utilized as an excellent source of natural health-promoting compounds.

According to the statistics from the Food and Agriculture Organization (FAO) of the United Nations, the world’s total production of sweet potatoes in 2020 was 105 million tons. Sweet potato is largely produced in Asia, followed by the African continent (Figure 1). Since sweet potatoes are grown in around 110 countries, the overwhelming of which are classified as developing nations. It is actually in developing countries that many farmers are highly dependent on root and tuber crops as sources of food, nutrition, and cash income. 

The introduction of crops with improved tolerances to pest, pathogens and viruses can significantly reduce environmental pollution, and at the same time minimize crop yield loss [1]. To further achieve sustainable agriculture, a reduction in the use of pesticides is extremely important. Meanwhile, the occurrence of extreme weather events has dramatically increased in the past decade, there is an urgent need to improve the resistance of major crops to abiotic stresses as well. For instance, drought is one of the major environmental factor causing abiotic stress and low productivity [2]. Severe drought restricts crop growth and significantly reduces yield worldwide. It is therefore essential that crops with drought tolerance traits are produced. However, in comparison with other important crop plants, sweet potato receives relatively little research attention. Although sweet potato has the advantage of being drought tolerant after establishment, dry matter accumulation and root tuber enlargement are inhibited under drought stress, which seriously hampers production. This issue contains seven research papers focusing on sweet potato in response to the biotic [1] and abiotic stresses [2,3,4,5,6,7]. Genetic engineering methods can be used to improve drought tolerance. Biotechnology offers great potential for improving disease, pest and stress resistance and nutritional quality of sweet potato. Conventional breeding can still be used for certain traits.

Sweet potato is a highly heterozygous, generally self-incompatible, polyploidy and vegetatively propagated, which poses numerous challenges for the conventional breeding. Research into the improvement of sweet potato has been neglected in favor of the more prestigious cereal or the other export cash crop. Nevertheless, sweet potato breeding needs to be accelerated to meet the needs of a growing world population, to promote sustainable agriculture and to address future environmental changes (Figure 2). The acceleration is highly reliant on the discoveries in gene functional studies. The release of the sweet potato reference genome has significantly facilitated the advance in sweet potato functional genomics. The ability to breed major crops has been greatly improved by advances in genomics, and sweet potato can benefit from the knowledge gained from the breeding of major crops, including the identification of genes that control key agronomic traits and the application of advanced breeding methods, etc. Genetic and genomic resources for sweet potato breeding are still lacking, which hamper further utilization of modern crop improvement tools such as genomic selection, genome editing, and high-throughput phenotyping. Genome sequencing has progressed, becoming more affordable and applicable to orphan crops. Limited understanding of molecular mechanisms behind certain bio-functions is available and remains to be better explored. The use of molecular breeding has great potential to increase nutritional and functional constituents in economically important sweet potato, thus allowing development in nutritionally improved varieties. This issue contains five papers about omics research to describe and discuss the mechanisms underlying the regulation of storage root development [5,8], nutrient substance [9], and important gene families [10,11]. With advances in genome sequencing, genome-based breeding methods have been widely used in major crops. These methods have shortened the breeding process and have improved selection efficiency. Theoretically, these genomic breeding methods could also be applied to or benefit sweet potato. To date, CRISPR/Cas has been utilized in over a dozen orphan crops with diverse genetic backgrounds, leading to novel alleles and beneficial phenotypes for breeders, growers, and consumers. There is a great potential for CRISPR/Cas-mediated gene editing in sweet potato improvement programs to solve a plethora of agricultural problems, especially impacting developing countries. In particular, precise editing of genes through the CRISPR-Cas9 approach could facilitate the development of improved sweet potato crop varieties. When applied to a new plant species, the CRISPR-Cas9 system often requires considerable optimization in terms of vector construction, transgene expression, tissue culture, transformation efficiency, heritable, and DNA-free plant genome editing due to its asexual propagation. Therefore, the use of genome editing in sweet potato is still a long way away.

Collectively, sweet potato fulfills basic roles in the global food system, which has fundamental implications for meeting food requirement, reducing poverty, and increasing food security. A concerted effort is urgently needed to advance the breeding of both orphan and major crops so that food security will be achieved and ultimately the livelihood of the population will be improved.

## Figures and Tables

**Figure 1:**
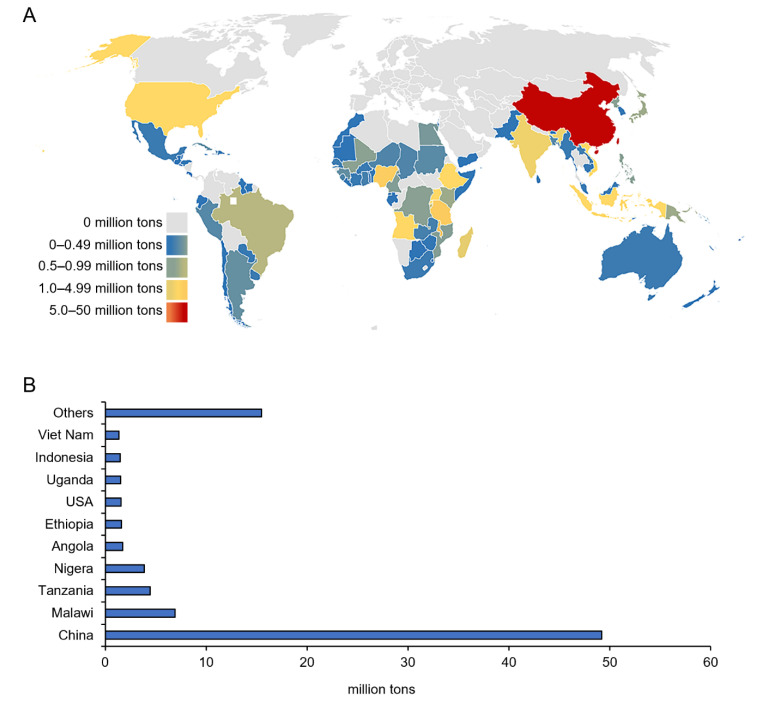
Sweet potato yield. (**A**) The distribution of sweet potato cultivation in the world. (**B**) Top 10 sweet potato producing countries worldwide in year 2020. China, Malawi, United Tanzania, Nigeria, Angola, Ethiopia, the United States of America (USA), Uganda, Indonesia, and Vietnam ranked among the top 10 in the world with only one developed country. Data source is FAO (2020).

**Figure 2:**
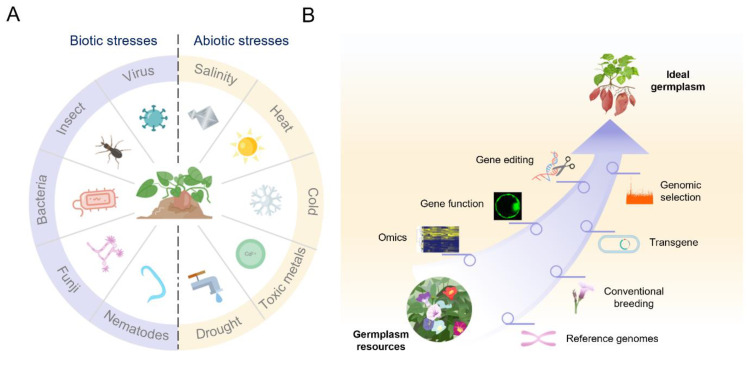
Proposed strategy for achieving sweet potato improvement. (**A**) Representation of biotic and abiotic stress factors that affect sweet potato production. (**B**) Harnessing the power of multi-approaches for genetic improvement in sweet potato. The use of traditional breeding and modern crop improvement tools, such as transgene, genomic selection, and gene editing, has made it possible to rapidly produce ideal germplasm.
